# Primary Atypical Lipomatous Tumor of the Orbit

**DOI:** 10.18502/jovr.v14i4.5479

**Published:** 2019-10-24

**Authors:** Mahmood Dhahir Al-Mendalawi

**Affiliations:** Department of Paediatrics, Al-Kindy College of Medicine, University of Baghdad, Baghdad, Iraq

Sir,

I read with interest the case report by Dworak et al on orbital atypical lipomatous tumor (ALT), a rare variety of liposarcoma, in an American patient.^[1]^ It is obvious that in addition to opportunistic infections, patients infected with human immunodeficiency virus (HIV) are also more susceptible to various types of tumors. The origin of these tumors is thought to be multifactorial, including immunosuppression, co-infection with oncogenic viruses, and life prolongation secondary to the use of antiretroviral therapy.^[2]^ Among these tumors, liposarcomas have been reported in HIV-positive patients.^[3,4]^ To my knowledge, HIV infection is a distressing health threat in the United States of America (USA). According to the available data, an estimated 1.1 million people aged ≥ 13 years were living with HIV infection in the USA at the end of 2015, including an estimated 162,500 (15%) persons with undiagnosed infections.^[5]^ I presume that HIV infection ought to be considered in the patient studied in the aforementioned case. Hence, testing of HIV status using the diagnostic panel of CD4 count and viral overload measurements was solicited in the studied patient. If that diagnostic panel was conducted and disclosed HIV infection, the case in question could surely be considered novel as HIV-associated orbital ALT has never been reported in the literature so far.

##  Financial Support and Sponsorship

Nil.

##  Conflicts of Interest

There are no conflicts of interest.
